# The Presence of Mycotoxins in Feed and Their Influence on Animal Health

**DOI:** 10.3390/toxins12100663

**Published:** 2020-10-20

**Authors:** Maciej T. Gajęcki, Magdalena Gajęcka, Łukasz Zielonka

**Affiliations:** Department of Veterinary Prevention and Feed Hygiene, Faculty of Veterinary Medicine, University of Warmia and Mazury in Olsztyn, Oczapowskiego 13/29, 10-718 Olsztyn, Poland; gajecki@uwm.edu.pl (M.T.G.); lukaszz@uwm.edu.pl (Ł.Z.)

Mycotoxins are secondary metabolites of fungi. They are commonly detected in food, feed [1,2,3,4], and feed additives [5]. The presence of mycotoxins in food and feed is influenced by numerous factors, including plant species and variety, region, temperature, moisture content, insect damage, storage conditions, and agricultural practices. Reisinger et al. [1] analyzed the prevalence of mycotoxins in maize silage, which is the key component of diets for dairy cattle. In addition to *Fusarium* mycotoxins zearalenone (ZEN), deoxynivalenol (DON), and nivalenol, maize silage in selected European countries also contains emodin, culmorin, enniatin, and beauvericin. The authors found that these mycotoxins compromised the gastrointestinal health of cattle during mixed mycotoxicosis and exerted unknown effects on the composition and function of the ruminal microbiota. Kemboi et al. [4] investigated the contamination of cattle feeds in various regions of Africa. They reported that local farmers made few attempts to minimize feed contamination with mycotoxins, which compromised the health quality of dairy products. African farmers lack the knowledge of how, when, and which detoxicants should be used, and the applied agents are often ineffective. Khoshal et al. [2] analyzed 524 samples of pig diets from around the world for the presence of 800 metabolites. Eighty-eight percent of the samples were contaminated with DON. Feeds also contained mycotoxins produced by fungi of the genera *Fusarium*, *Aspergillus*, *Penicillium*, and *Alternaria*, and their in vitro toxicity was similar to that of DON. The identified compounds were arranged in the following order based on their toxicity: apicidin > enniatin A1 > DON > beauvericin > enniatin B > enniatin B1 > emodin > aurofusarin. An in vivo test also revealed that the presence of additional metabolites did not increase the overall toxicity. Witaszak et al. [3] examined the presence of mycotoxins in dry food for dogs and cats. Their research was motivated by the fact that cereals are a major ingredient in dry food for companion animals. The authors concluded that more effective solutions are needed to reduce the concentrations of mycotoxins in plant materials and animal feeds, and, more importantly, that the proportion of plant materials in the diets of domesticated predators should be decreased. The use of by-products from medicinal fungi in the production of animal feeds also poses a problem [5]. These by-products contain compounds such as adenosine, cordycepin, and pentostatin, as well as substances with cytotoxic and neurotoxic properties. The authors found that *Cordyceps* fungi produced large amounts of unidentified secondary metabolites which were secreted by biosynthetic gene clusters (BGC). These compounds could also be applied in the pharmaceutical industry.

At present, risk evaluations focus mainly on mycotoxins’ ability to modify the metabolic profile and their proinflammatory, mutagenic, cytotoxic, and potential carcinogenic effects [6,7,8,9]. Rykaczewska et al. [6] reported that pre-pubertal gilts responded differently to ZEN administered at the lowest-observed-adverse-effect level (LOAEL), no-observed-adverse-effect level (NOAEL), and minimal anticipated biological effect level (MABEL) doses. One of the differences was the fact that the proportion of β-ZEL increased in the group of ZEN metabolites. Different responses were observed in female pigs from other age groups. Beta-ZEL exerts varied effects—it induces a minor increase in body weight gain, but also slows down the sexual maturation in gilts. The ZEN levels are initially very low, and its metabolites are not detected in the blood serum (in particular under exposure to the MABEL dose), which confirms that gilts have a high physiological demand for exogenous estrogen-like substances. These substances are readily utilized by pre-pubertal gilts. When administered at higher doses, excess “free” ZEN plays other, not always beneficial, roles, and it can lead to ovarian atrophy or silent heat. The levels of estradiol (E_2_) and “free” ZEN increase proportionally to the ZEN dose, which suppresses the concentrations of progesterone (P_4_) and testosterone (T). Zielonka et al. [8] analyzed the concentrations of selected steroid hormones (E_2_, P_4_, and T) in premenopausal pigs administered ZEN at 20 or 40 µg/kg BW for 48 days, and observed that (i) the concentrations of ZEN in the peripheral blood were very low and highly varied on different days of exposure, and their diagnostic value was difficult to determine; (ii) experimentally induced hyperestrogenism or “supraphysiological hormone levels” contributed to a minor increase in the total E_2_ levels (which could intensify proliferation processes) and suppressed T concentrations; (iii) the results can be extrapolated to indicate that the analyzed doses of ZEN produced varied responses, where a lower dose probably exerted stimulatory/adaptive effects and a higher dose inhibited physiological processes in the studied animals. Wang et al. [7] confirmed the proinflammatory effects of mycotoxins in vitro. They found that nitric oxide (NO) activity and the relative expression of *iNOS* mRNA increased with a rise in DON dose, and the relative expression of the *COX*-2 gene, which was identical to that of the induced enzyme, also increased in porcine intestinal epithelial cells (IPEC-J2). Intestinal epithelial cells were stimulated by DON to produce an inflammatory response. However, the NF-κB pathway is a potential pathogenic factor which, when activated incorrectly or in excessive amounts, exerts adverse effects on cells. Obremski et al. [9] arrived at similar conclusions in a study investigating the effects of very low ZEN doses on (i) the secretion of proinflammatory cytokines, (ii) the secretion of anti-inflammatory and regulatory cytokines, (iii) oxidative stress markers, and (iv) basic metabolic markers. They found that low ZEN doses induced an inflammatory response. The proinflammatory properties of ZEN and intensified oxidative stress can impair intestinal epithelial function, as demonstrated by oxidative stress markers such as the biochemical changes associated with the metabolism of sugars (intensified glycolysis) and amino acids (proline).

Recent research has demonstrated that mycotoxins exert adverse effects on sensitive structures in the intestines [9,10] and target tissues/organs such as the liver [11]. In a study by Śliżewska et al. [10], ochratoxin A (OTA) present in low concentrations in turkey feed caused body weight loss, depression, and paralysis, leading to a decrease in the mobility and energy levels of birds. The above symptoms were accompanied by catarrhal inflammation of the gastrointestinal mucosa and local ecchymoses on the liver and kidney surface. To prevent the adverse effects of OTA, turkey diets were supplemented with three synbiotic preparations. The tested synbiotics contributed to an increase in the counts of beneficial bacteria and a decrease in the counts of pathogenic gut microbiota. They also increased the activity of α-glucosidase and α-galactosidase while decreasing the activity of fecal enzymes, which exerted a beneficial influence on the health status of turkeys and improved their body weight gains. Skiepko et al. [11] found that low doses of ZEN and DON in feed induced changes in the histology and ultrastructure of the liver in prepubertal gilts, including (i) an increase in the thickness of the perilobular connective tissue and lobe penetration by connective tissue; (ii) an increase in the histology activity index; (iii) the widening of the liver sinusoids; (iv) transient changes in the glycogen content; (v) the increased accumulation of iron in hepatocytes; (vi) changes in the organization of the endoplasmic reticulum in hepatocytes; (vii) changes in the morphology of Kupffer–Browicz cells. The above observations suggest that low doses of mycotoxins administered individually or in combination, even for short periods of time, affected the liver morphology. Another study [12] postulated that mycotoxins could exert adverse effects on the enteric nervous system (ENS) and that their influence should be analyzed in greater detail. The ENS plays a key role in the regulation of most gastrointestinal functions; it participates in adaptive processes and defense mechanisms and acts as one of the first barriers against pathogens and toxins in feed materials. Mycotoxins can exert multidirectional effects depending on their chemical structure, the investigated mammalian species, and the type of nerve fiber bundles and segment of the gastrointestinal tract affected. Mycotoxins can affect the size and morphology of intestinal nerve fibers and the neurochemical characteristics of enteric neurons. These changes are probably induced by adaptive and defense responses that promote homeostasis. The changes observed in the ENS are often the first symptoms of contamination with low mycotoxin doses.

A review of the presented research studies indicates that reference biomarkers should be developed to support quick evaluations of animal health (in particular, the gastrointestinal tract and the accompanying tissues) during ongoing mycotoxicosis, regardless of the dose of the parent compound and its metabolites. The above applies to livestock as well as companion animals that are fed monotonic diets for long periods of time.
